# Predicting the immunity landscape and prognosis with an NCLs signature in liver hepatocellular carcinoma

**DOI:** 10.1371/journal.pone.0298775

**Published:** 2024-04-25

**Authors:** Zhangxin Ji, Chenxu Zhang, Jingjing Yuan, Qing He, Xinyu Zhang, Dongmei Yang, Na Xu, Jun Chu

**Affiliations:** 1 Key Laboratory of Xin’an Medicine, Ministry of Education, Anhui University of Chinese Medicine, Hefei, Anhui, PR China; 2 School of Graduate, Anhui University of Chinese Medicine, Hefei, Anhui, PR China; 3 Research and Technology Center, Anhui University of Chinese Medicine, Hefei, Anhui, PR China; 4 State Key Laboratory of Tea Plant Biology and Utilization, School of Tea & Food Science and International Joint Laboratory on Tea Chemistry and Health Effects of Ministry of Education, Anhui Agricultural University, Hefei, Anhui, PR China; 5 Institute of Surgery, Anhui Academy of Chinese Medicine, Anhui University of Chinese Medicine, Hefei, Anhui, PR China; Amity University Rajasthan, INDIA

## Abstract

**Background:**

Activated neutrophils release depolymerized chromatin and protein particles into the extracellular space, forming reticular Neutrophil Extracellular Traps (NETs). This process is accompanied by programmed inflammatory cell death of neutrophils, known as NETosis. Previous reports have demonstrated that NETosis plays a significant role in immune resistance and microenvironmental regulation in cancer. This study sought to characterize the function and molecular mechanism of NETosis-correlated long non-coding RNAs (NCLs) in the prognostic treatment of liver hepatocellular carcinoma (LIHC).

**Methods:**

We obtained the transcriptomic and clinical data from The Cancer Genome Atlas (TCGA) and evaluated the expression of NCLs in LIHC. A prognostic signature of NCLs was constructed using Cox and Last Absolute Shrinkage and Selection Operator (Lasso) regression, while the accuracy of model was validated by the ROC curves and nomogram, etc. In addition, we analyzed the associations between NCLs and oncogenic mutation, immune infiltration and evasion. Finally, LIHC patients were classified into four subgroups based on consensus cluster analysis, and drug sensitivity was predicted.

**Results:**

After screening, we established a risk model combining 5 hub-NCLs and demonstrated its reliability. Independence checks suggest that the model may serve as an independent predictor of LIHC prognosis. Enrichment analysis revealed a concentration of immune-related pathways in the high-risk group. Immune infiltration indicates that immunotherapy could be more effective in the low-risk group. Upon consistent cluster analysis, cluster subgroup 4 presented a better prognosis. Sensitivity tests showed the distinctions in therapeutic effectiveness among various drugs in different subgroups.

**Conclusion:**

Overall, we have developed a prognostic signature that can discriminate different LIHC subgroups through the 5 selected NCLs, with the objective of providing LIHC patients a more precise, personalized treatment regimen.

## 1. Introduction

Liver hepatocellular carcinoma (LIHC) is a prevalent global health threat. The primary risk factors for LIHC include chronic hepatitis, alcoholic liver disease, metabolic liver disease (particularly non-alcoholic fatty liver disease) and dietary toxins [1, 2]. Although there are several therapeutic options available in contemporary medicine, which consist of surgical resection, liver transplantation and percutaneous ablation, survival remains poor for patients who present with advanced cancer recurrence and metastases [3]. The immune regulation of body plays an integral role in the progression of LIHC. In order to advance treatment to the early stages of tumors, the chief challenge with LIHC immune checkpoint inhibitor therapies is now the identification and validation of more predictive biomarkers [4]. Therefore, it is critical to identify additional effective biomarkers to improve the therapeutic outlook for LIHC patients.

Neutrophils are essential for the tumor immune resistance. Neutrophil Extracellular Traps (NETs) are reticular DNA structures wrapped by nuclear proteins (e.g. histones, granulins and cytoplasmic proteins), that are discharged from activated neutrophils [5]. The accumulation of NETs is paralleled with neutrophil death, which is a novel form of programmed cell death that diverges from apoptosis or necrosis, known as NETosis [6]. Numerous evidences have revealed that NETosis is closely related to the growth, development and metastasis of various cancer. In particular, the expression of PAD4 in neutrophils promotes the citrullination of histones in a calcium-dependent environment, which drives NETosis and contributes to primary tumor growth and the formation of Cancer-Associated Thrombosis (CAT) [7]. Additionally, it has been discovered that neutrophils and Circulating Tumor Cells (CTCs) have a reciprocal relationship. Surgically activated platelets produce NETs that enhance the capture and distant metastasis of CTCs [8]. Nevertheless, researches on NETosis in LIHC are limited, and identifying emerging NETosis-related biomarkers is of interest for the prognosis of LIHC patients.

RNAs longer than 200 nucleotides that are not involved in protein coding, known as long non-coding RNAs (lncRNAs) [9]. Abnormalities in their expression or function of lncRNAs are tightly linked to the development of many diseases, including cancer [10, 11]. A recent study has identified lncRNA RP11-386G11.10 as a new oncogenic lncRNA that is stringently correlated with adverse progress of liver cancer [12]. Meanwhile, lncRNAs have been shown to be involved in the regulation of NETosis [13]. However, there are no sufficient studies on NETosis-correlated LncRNAs (NCLs) markers to accurately predict the prognosis of patients with LIHC.

**Fig 1:**
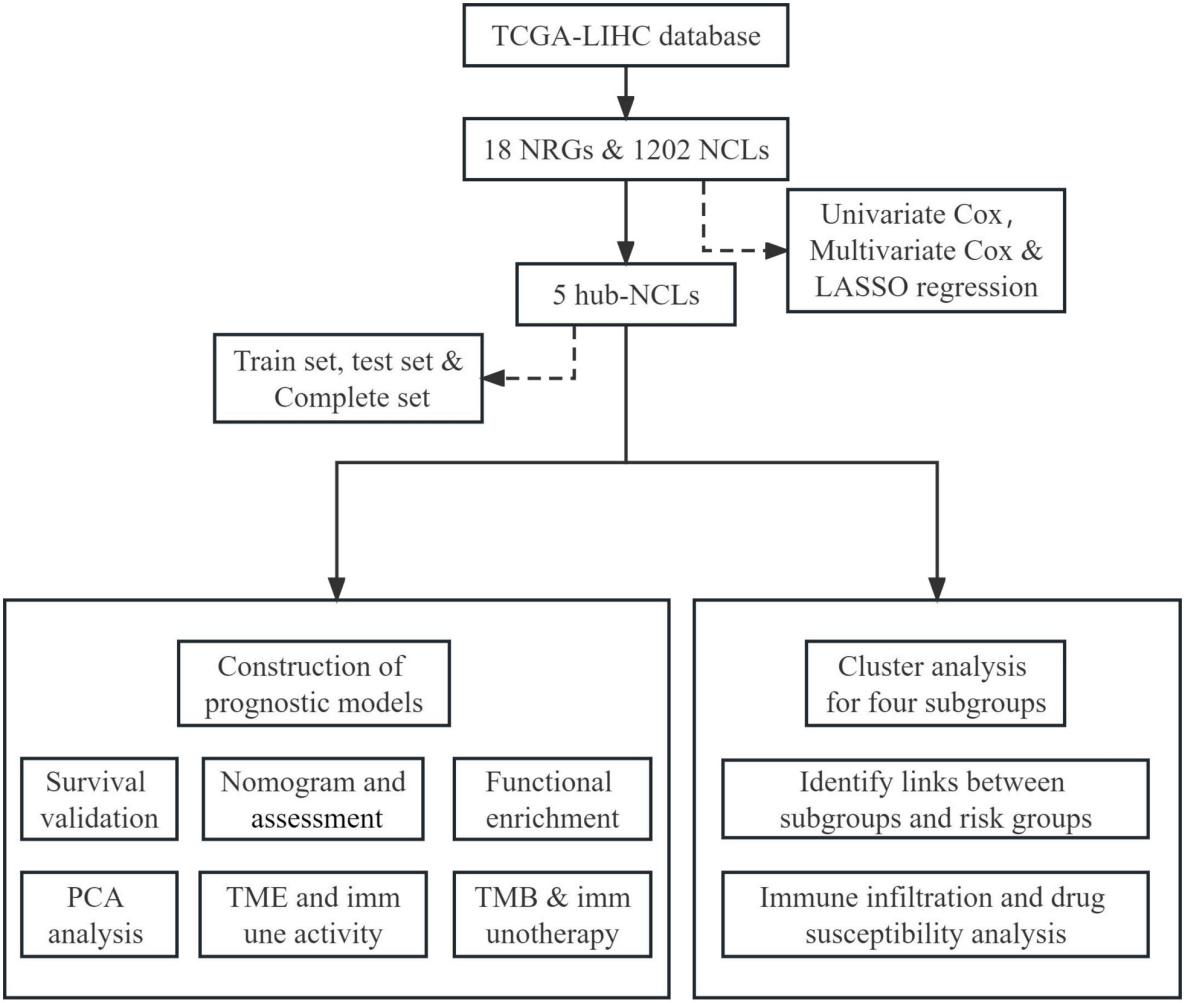
Workflow diagram for this research.

The present study aims to investigate NCLs in LIHC to understand the regulatory mechanisms and signaling pathways underlying the NETosis in this malignancy. Moreover, we intend to establish a novel signature of NCLs that may aid in prognostic testing as well as clinical pharmacotherapy of LIHC patients. Following Fig 1 depicts our research flow.

## 2. Materials and methods

### 2.1 Patient details acquired

Transcriptomic profiles and clinical records for LIHC patients (including 50 normal and 374 tumor samples) were derived from The Cancer Genome Atlas (TCGA) database (https://portal.gdc.cancer.gov/). Clinicopathologic attributes mainly included age, gender, tumor grade, stage, survival time, and survival status. The series data were systematically differentiated and processed between mRNA and lncRNA by means of Strawberry Perl (https://strawberryperl.com/). Additionally, we gathered the Simple Nucleotide Variation (SNV) data to calculate the tumor mutational burden of LIHC.

### 2.2 NCLs identified

Through literature search, we collected 18 NETosis-related genes from recent studies, as shown in S1 Table. The "limma" package for R software (version 4.3.2, https://www.rproject.org/) was applied to extract the expression of relevant genes, and Pearson correlation analysis was performed between NRGs and lncRNAs (R > 0.4 & P < 0.001) [14]. Additionally, packages such as "dplyr", "ggalluvial", and "ggplot2" were utilized to analyze NCLs and survival data for LIHC patients.

### 2.3 Establishment of prognostic signature

R packages "survival", "caret", "glmnet", "survminer ", and "timeROC" were employed to process prognostically relevant lncRNAs and construct prognostic models. The selected lncRNAs were combined with clinical information about the disease and the LIHC samples from TCGA were then randomized into two groups, a train group for constructing the signature and a test group as well as the whole dataset for validating the signature [15]. Then, to prevent potential overfitting in the building of the prognostic model, we utilized the Least Absolute Shrinkage and Selection Operator (Lasso) regression to filter for risk-related lncRNAs. The risk score was computed applying this following formula:

$$RS\left(risk\;score\right)=\sum \left[Exp\left(lncRNA\right)\times coef\left(lncRNA\right)\right].$$

Based on the median score, LIHC patients were separated into low and high risk sections. In the next step, with the "survival", "regplot", "rms", and "survcomp" packages, a nomogram was constructed to predict survival in patients with LIHC using clinical parameters and risk scores based on multifactorial Cox regression results. The precision and practicality of the model were evaluated via the Area Under Curve (AUC), Conformance index (C-index), and survival curve, with the "rms", "pec", "survival", "survminer", "timeROC" packages. Meanwhile, we assessed the expression and survival of individual lncRNAs in LIHC patients utilizing the Ualcan database (https://ualcan.path.uab.edu/).

### 2.4 Principal component analysis and enrichment analysis

The processed lncRNAs underwent Principle Component Analysis (PCA) using the "limma" and "scatterplot3d" packages to determine their ability to differentiate the risk groups of LIHC. Afterwards, Gene Ontology (GO), Kyoto Encyclopedia of Genes and Genomes (KEGG), and Gene Set Enrichment Analysis (GSEA) enrichment analyses were performed using the "clusterProfiler" and "enrichment plot" packages, with a threshold of FDR < 0.05.

### 2.5 Immune infiltration analysis

We calculated stromal, immune and estimated scores for patients with LIHC using the "limma" and "estimate" packages, comparing the differences between the high- and low- risk groups. The relationship between risk scores and tumor-infiltrating immune cells was then assessed by means of XCELL, TIMER, QUANTISEQ, etc. The Wilcoxon analysis was applied to compare the differences in the levels of immune infiltrating cells. Spearman analysis was performed to examine the connection between the risk score and immune cells. Finally, the correlation between risk score and immunized function was investigated by the single sample GSEA (ssGSEA) and "GSVA" packages. These analyses enable the validation of the effectiveness of immune checkpoint inhibitors in LIHC.

### 2.6 Tumor mutation burden (TMB) and immune evasion

Defined as the total number of somatic variants detected per million bases, TMB is gaining attention as an emerging biomarker for predicting the efficacy of tumor immunotherapy. In the present study, somatic cell mutation data was obtained from TCGA. Using the "maftools" package, the distribution of mutation profiles in the high- and low-risk groups were visualized. We generated bubble plots for correlation analysis between immune cells and prognostic models using the R packages "scales", "ggplot2", "ggtext", "tidyverse", and "ggpubr". Survival curves for both TMB subtypes were plotted using the Kaplan-Meier (KM) method. After acquiring immune dysfunction ratings from the Tumor Immune Dysfunction and Exclusion (TIDE) online database (http://tide.dfci.harvard.edu/), variations in TIDE scores between high- and low- risk categories in LIHC samples were analyzed with the packages "limma" and "ggpubr".

### 2.7 Consensus clustering

The "ConsensusClusterPlus" package was used to perform consensus clustering based on the expression of lncRNAs. To compare the prognostic and drug sensitivity discrepancies, PCA, t-distributed Stochastic Neighbour Embedding (t-SNE), KM survival analysis, and immune correlation tests were performed on clustered LIHC patients using the "Rtsne" and "ggplot2" packages.

## 3. Results

### 3.1 Identification of NCLs in LIHC

**Fig 2:**
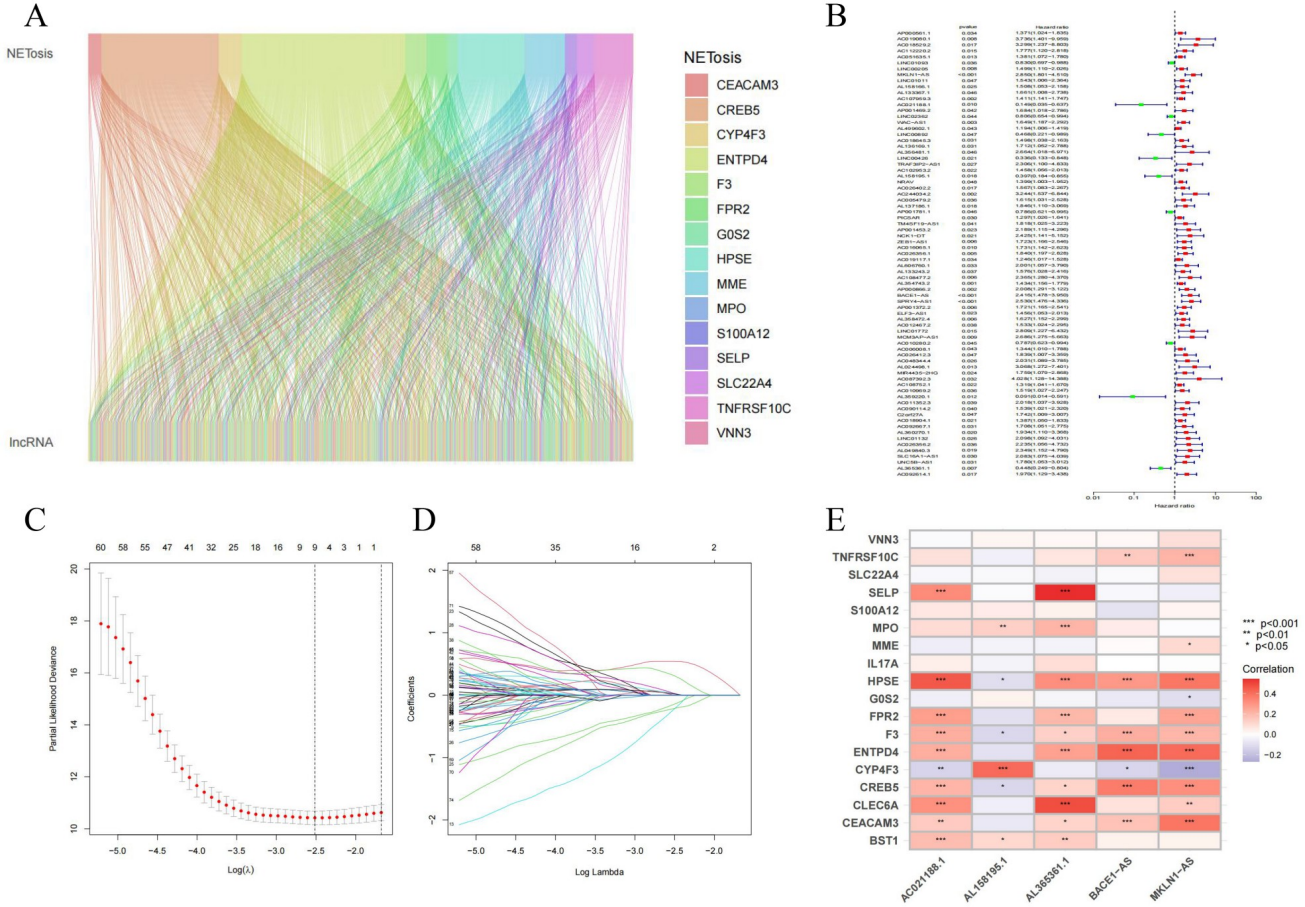
Modelling to determine the prognostic value of NCLs in LIHC. **(A)** Co-expression analysis of NETosis with lncRNAs. **(B)** Forest plot findings from univariate Cox regression analysis. **(C, D)** Lasso analysis and Cox regression for the construction of models. **(E)** Correlation analysis of lncRNAs with NETosis genes.

A total of 1202 NCLs were identified in LIHC (Fig 2A; |Pearson R| > 0.4, p < 0.001). We applied univariate cox analysis to determine the prognostically significant lncRNAs among these 1202 NCLs. The results yielded a total of 75 lncRNAs associated with the prognosis of NETosis (Fig 2B). Subsequently Cox regression and Lasso analysis were performed to construct a prognostic signature of relevant lncRNAs in LIHC (Fig 2C and 2D). In addition, The final risk model obtained from this multi-cox analysis consisted of 5 hub NCLs (Fig 2E). The final risk score calculation formula was constructed as AC021188.1 × (-1.75805398351244) + AL158195.1 × (-0.839092908812043) + BACE1-AS × (1.05514888228153) + AL365361.1 × (-0.543082104068263) + MKLN1-AS × (0.73668113446929).

### 3.2 Tests of NETosis-related prognostic signature

**Fig 3:**
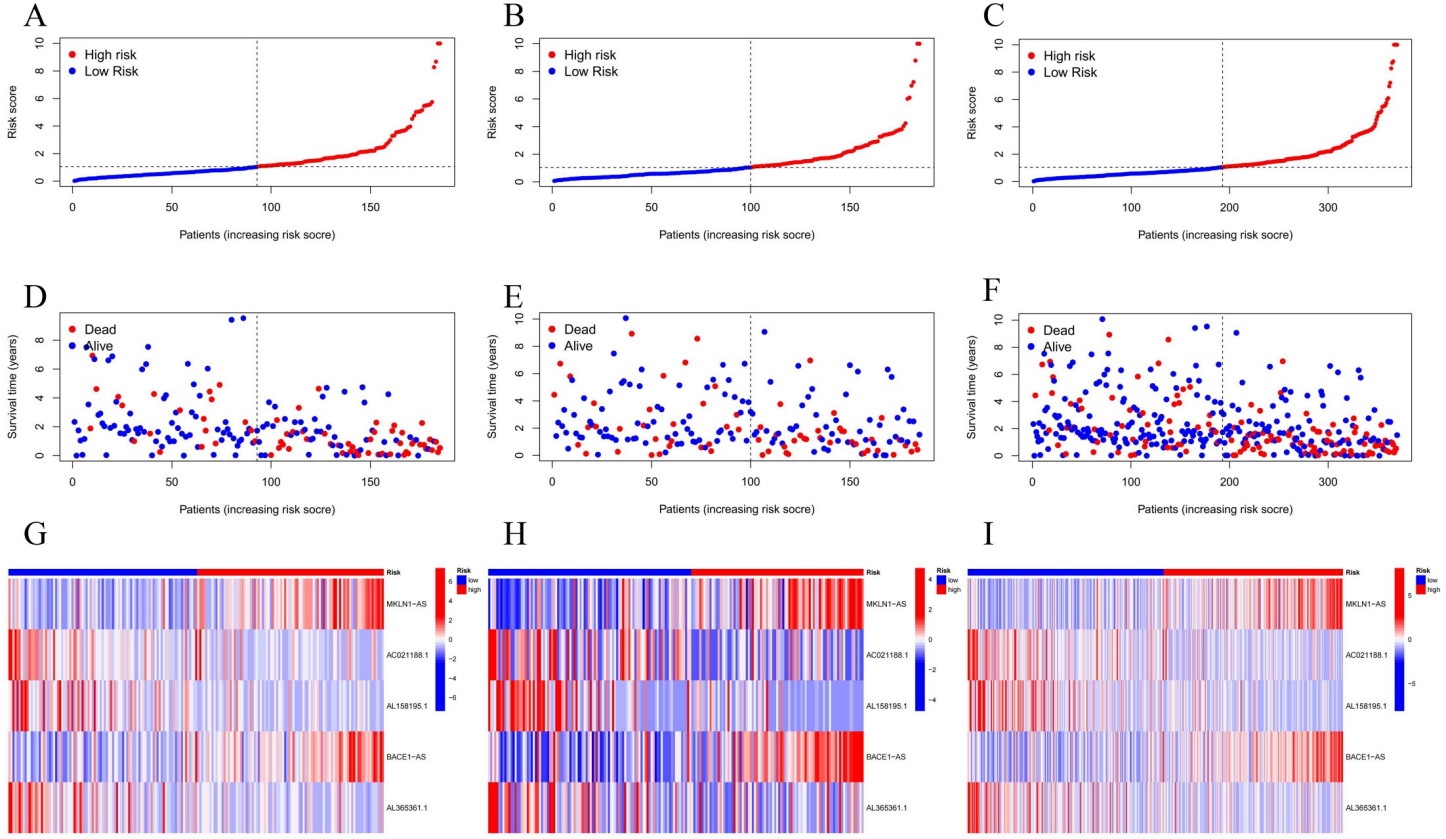
Evaluating the potency of NCLs in different subsets of the LIHC. Risk scores **(A-C)**, survival status **(D-F)**, and gene expression **(G-I)** for prognostic signature are demonstrated. From left to right, the train set, test set, and complete set.

**Fig 4:**
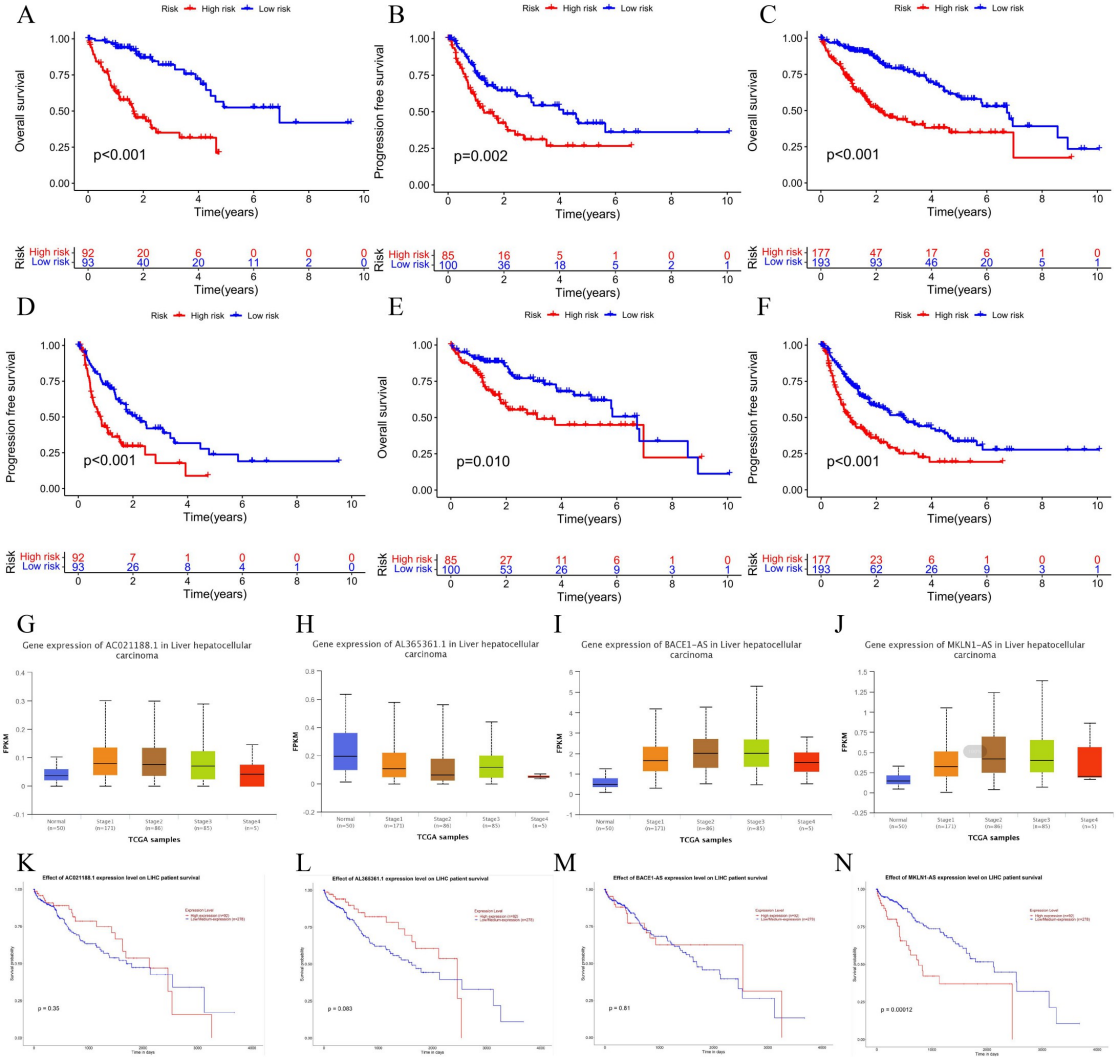
Survival analysis in high and low risk groups and hub lncRNA expression in LIHC. KM curves for OS **(A-C)** and PFS **(D-F)** of distinct risk groups in the train, test and all set. AC021188.1, AL158195.1, BACE1-AS, MKLN1-AS differences in expression **(G-J)** and survival **(K-N)** in LIHC.

According to our prognostic model, LIHC cases in TCGA were categorized into high- and low-risk subtypes, whereas risk scores, survival situation and expression levels of screened lncRNA for the train set, test and plenary set are shown in Fig 3A–3I. The findings of the survival study demonstrated that the Overall Survival (OS) and Progression Free Survival (PFS) of our predicted high-risk LIHC patients were consistently and significantly worse (Fig 4A–4F). Interestingly, despite missing data related to AL158195.1, analysis for individual NCLs showed high expression of MKLN1-AS in samples from all stages of LIHC, and the higher expression was accompanied by a significantly poorer prognosis (Fig 4G–4N).

### 3.3 Evaluation of prognostic signatures of NCLs

**Fig 5:**
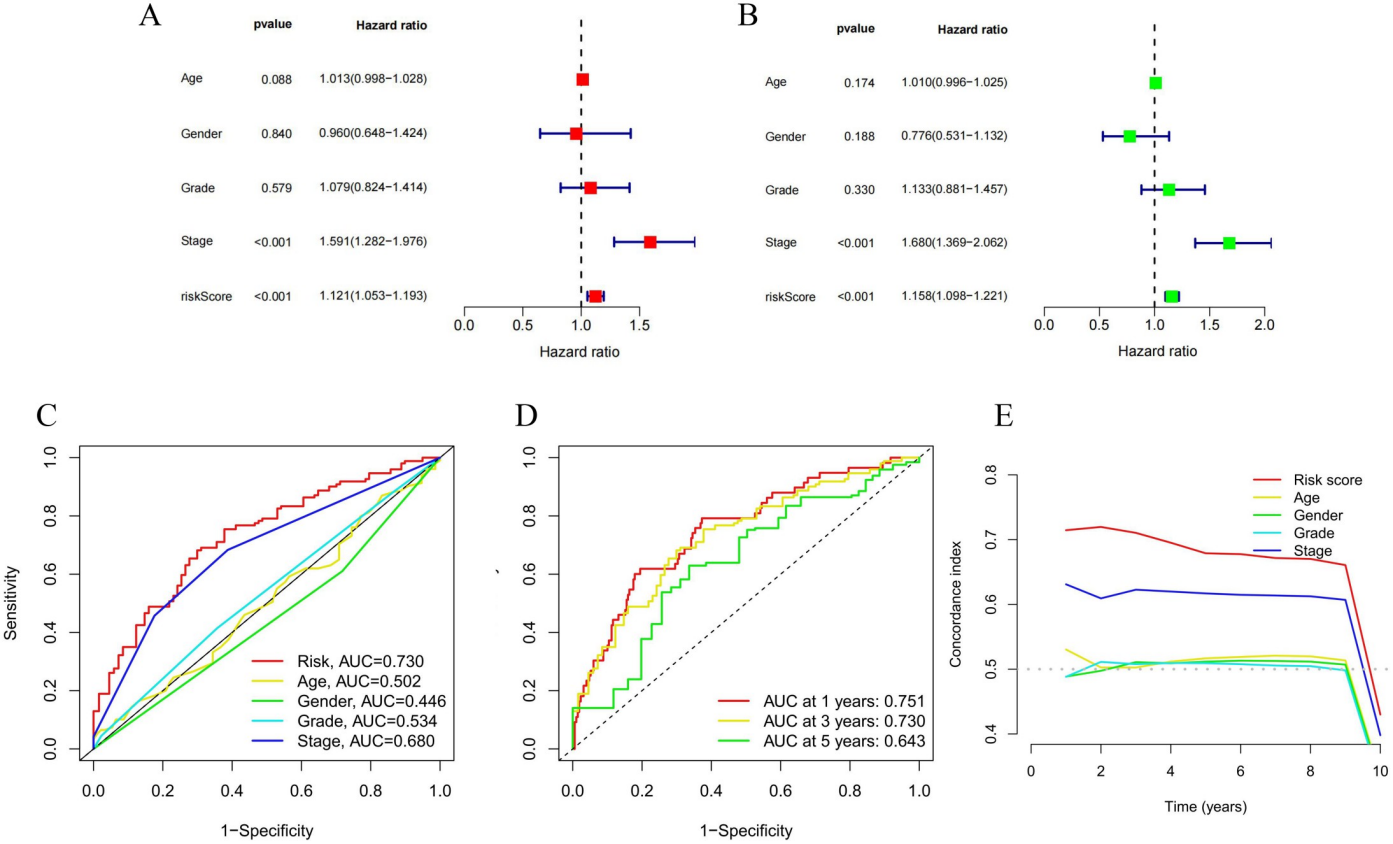
Validation of the predictive capability of risk models. **(A, B)** Univariate Cox regression analysis combining clinical characteristics and risk scores. **(C, D)** ROC curves for clinical characteristics as well as risk scores & survival ROC curves within different years. **(E)** Concordance indexes of prognostic characteristics.

**Fig 6:**
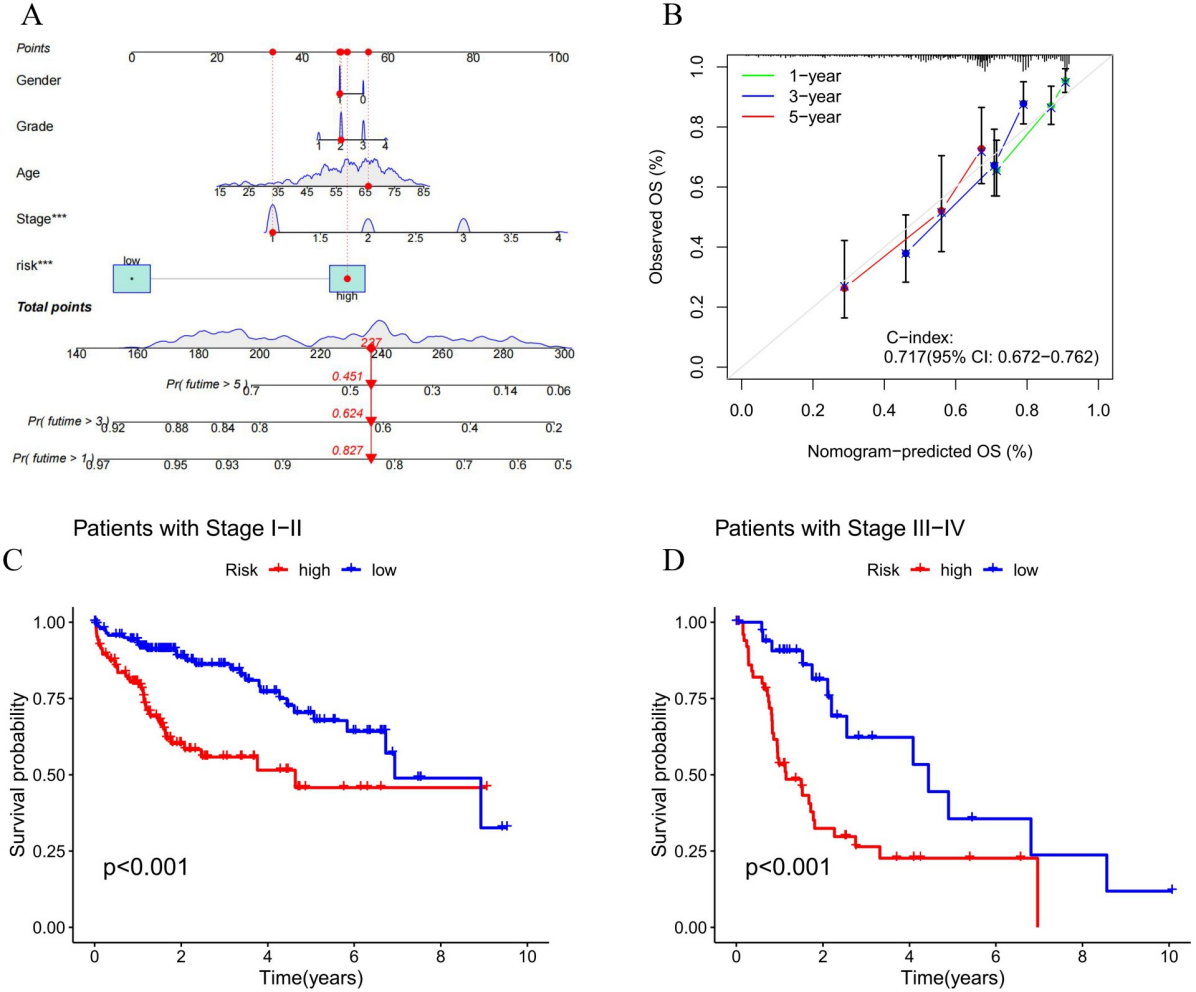
Predicting nomograms and evaluating survival in substages. **(A, B)** Calculation of a nomogram of risk scores to forecast the probability of survival in patients with LIHC. **(C, D)** High- and low-risk survival in different stages of patients.

We acquired univariate and multivariate independent prognostic analyses to better validate the accuracy of our model. Age, clinical grade and stage, and risk score were found to be independent predictive variables for LIHC (Fig 5A and 5B). Further, compared with other prognostic factors, the ROC curve of our model score had largest AUC value of 0.730, and the AUCs of the risk score for 1-, 3-, and 5-year were 0.751, 0.730, and 0.643, respectively (Fig 5C and 5D). The results of C-index curves within ten years also corroborated that the model constructed based on the NCLs could better predict the survival status of LIHC (Fig 5E). In addition, we created a nomogram by combining clinical traits and risk scores (Fig 6A). As we predicted, the nomogram performed effectively in the calibration curves of 1-, 3-, and 5-year OS (Fig 6B), and the samples with higher risk scores of LIHC had poorer OS in both the stage I-II and III-IV groups, with good significance of the results (Fig 6C and 6D).

### 3.4 Principal component analysis and enrichment analysis

**Fig 7:**
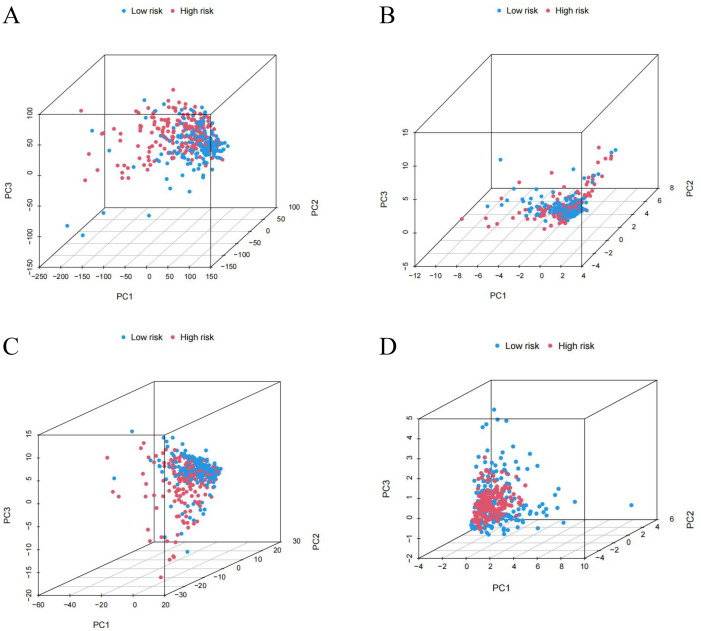
PCA illustrates that risk model can accurately classify high and low risk categories. **(A)** All genes **(B)** NETosis genes **(C)** NETosis LncRNA **(D)** Risk LncRNA.

**Fig 8:**
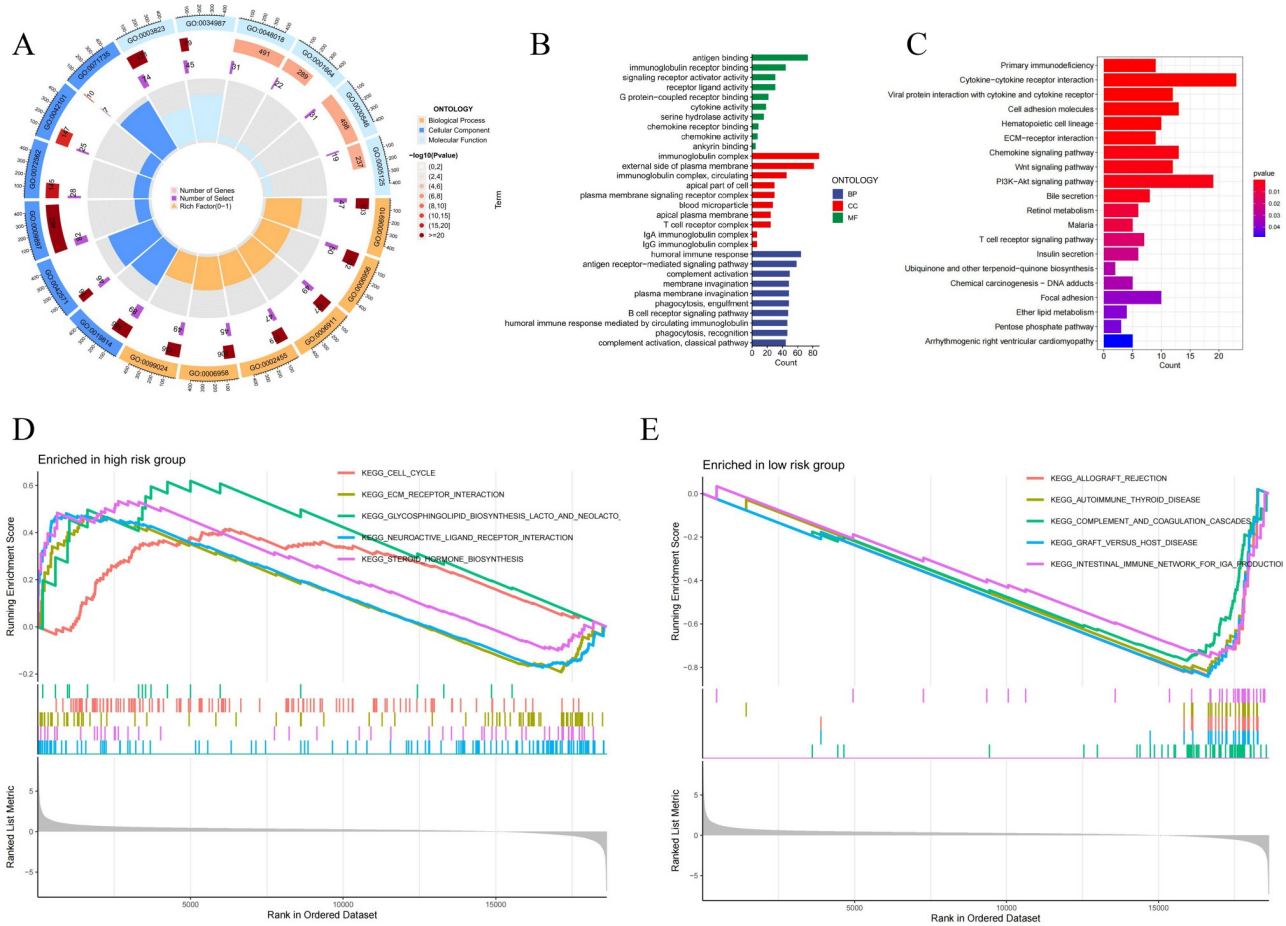
Enrichment analyses. GO **(A, B)** and KEGG **(C)** enrichment, along with multiple GSEA analyses **(D, E)**.

By performing principal component analysis, we found that NCLs could better distinguish LIHC samples into high- and low-risk groups (Fig 7A–7D). To identify functional differences between high- and low-risk groups, we also performed GO and KEGG enrichment analyses (Fig 8A–8C). The GO outcomes indicated that NCLs were tightly associated with antigen binding, immunoglobulin complex, and humoral immune response. While KEGG analysis showed that NCLs were correlated with primary immunodeficiency, PI3K-Akt and Wnt signaling pathway, suggesting that NCLs may be broadly engaged in the organism’s immunological response. Moreover, Fig 8D and 8E illustrated the five pathways that were mainly enriched in each of the high-and low-risk groups.

### 3.5 Tumor microenvironment and immunomodulation

**Fig 9:**
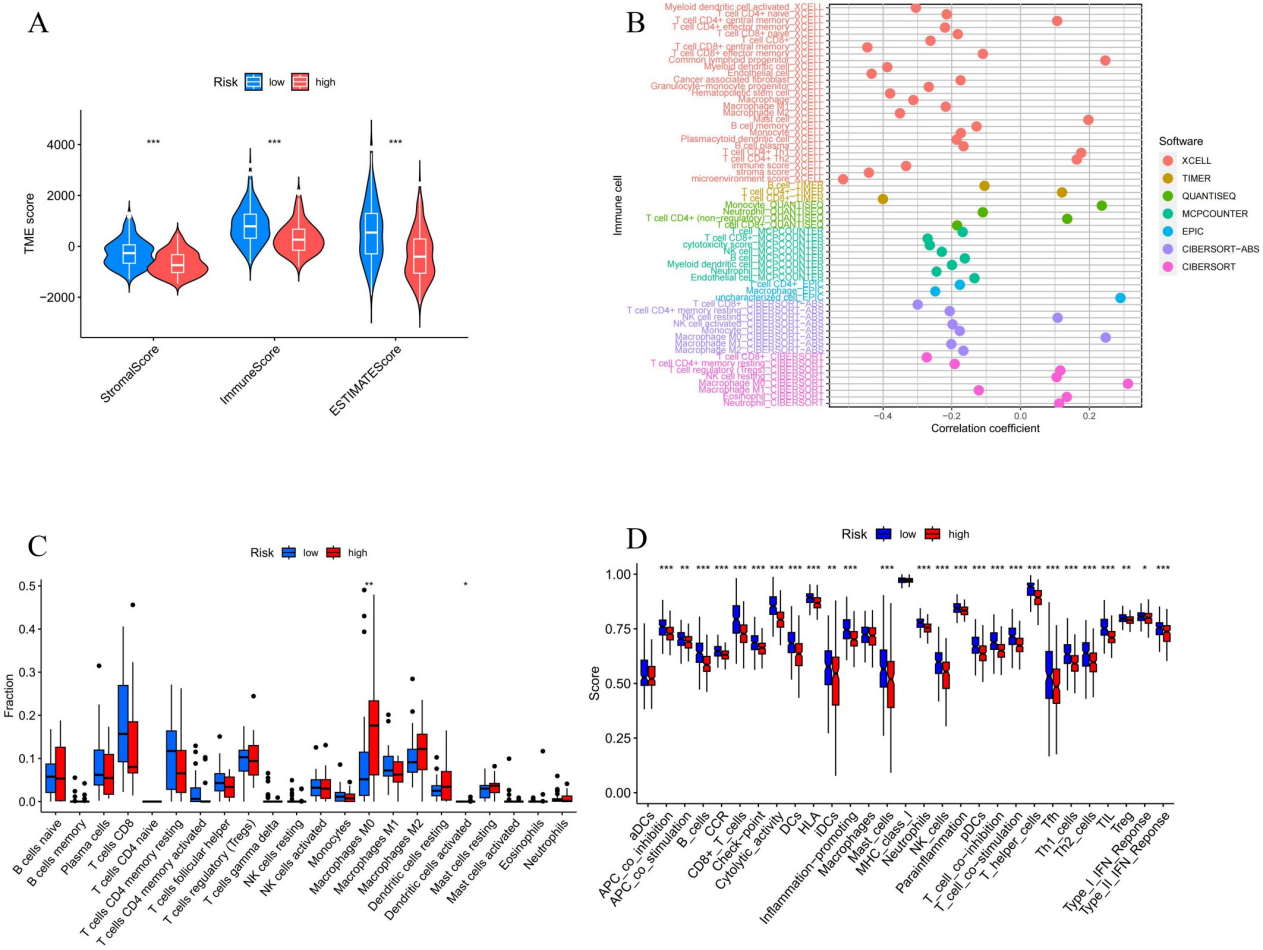
Investigating tumor immunity to LIHC in the NETosis risk model. **(A)** Differences in the immune microenvironment between high- and low-risk samples. **(B)** Immune cell correlation analysis in different software. **(C, D)** Differences in immune cells and immune cell-related functions between the high-/low-risk samples.

Since our research indicated that the high-threat group was connected to several immune-related processes, and after further clarifying the specific relationship between organismal immunity and prognostic models, the tumor microenvironment (TME) results indicated that LIHC patients with high-threat had lower stromal, immune, and estimate scores compared to the low-threat group (Fig 9A). Specifically, Fig 9B explained that patients with higher risk scores exhibited a negative correlation with T-cells CD8+, CD4+, B-cells, memory B-cells, plasma B-cells, NK-cells, myeloid dendritic cells, and a negative correlation with Mast-cells, common lymphoid progenitor cells. Relatively, Resting NK cells, macrophage M0 were positively correlated. In addition, Fig 9C also showed a significant rising expression of macrophage M0 in the high-risk group. Further, studies on immune-related functions demonstrated that APC regulation, CCR activity, Cytolytic activity, Inflammation-promoting, Parainflammation, and TIL response scores were generally and significantly lower in the high-risk group (Fig 9D).

### 3.6 Mutation and immune escape

**Fig 10:**
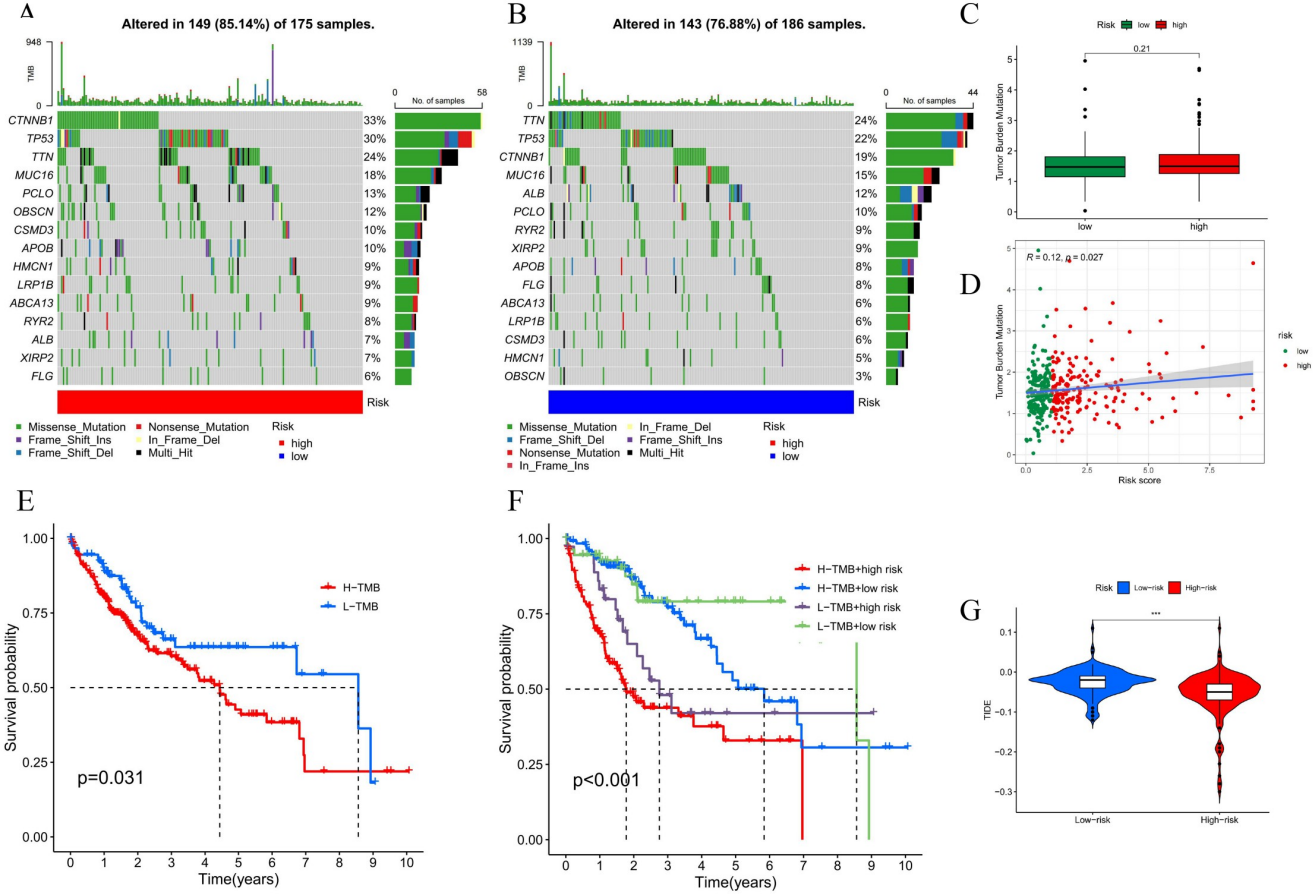
Analysis of somatic cell mutations and tumor mutation burden and immune escape. **(A, B)** Waterfall plot of mutations between high- and low- risk specimens. **(C, D)** TMB and the correlation of TMB with risk scores. **(E, F)** Differentiate survival curves for TMBs and TMBs with risk clusters.

To explore the mutational differences from one risk group to another, we investigated the TCGA cohort’s somatic mutation data for LIHC. The mutation probability was greater in the high-threat group, and the five genes with the highest mutation frequencies were CTNNB1, TP53, TTN, MUC16, and PCLO (Fig 10A and 10B). The TMB was calculated based on the mutation frequencies, although the statistical differences between two groups were not significant (P>0.05) (Fig 10C and 10D).The results of the survival analysis showed that patients with higher mutation frequencies had a reduced likelihood of surviving, and this effect was exacerbated by the high-risk groups differentiated according to the model (Fig 10E and 10F). In addition, the immune escape findings demonstrated that the high-risk group was accompanied by a lower TIDE score, which predicted a poorer benefit from receiving immunotherapy (Fig 10G).

### 3.7 Consensus clustering

**Fig 11:**
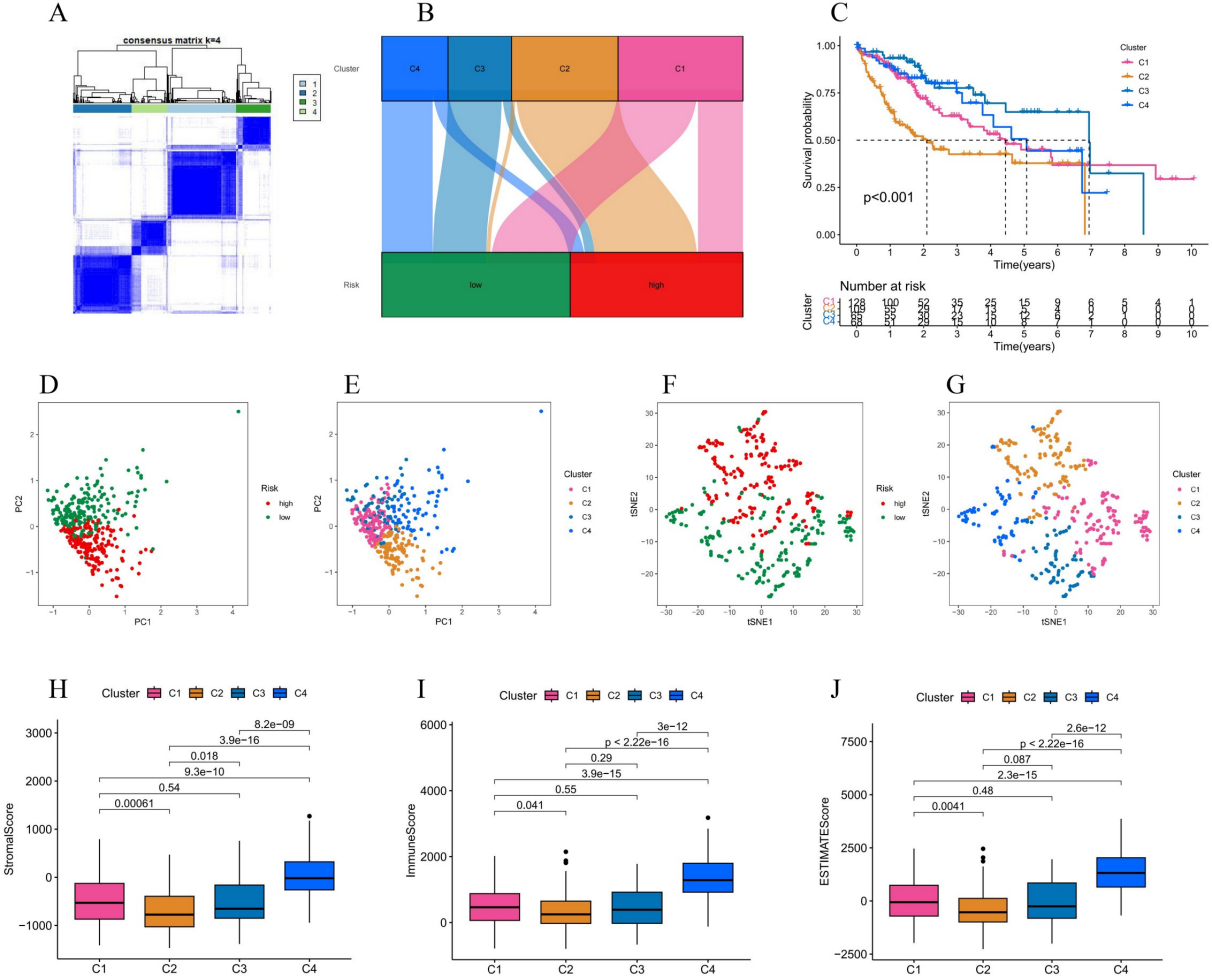
Secondary clustering of LIHC patients in TCGA based on the expression of NCLs. **(A)** Tumor samples were divided into four subgroups. **(B)** Correlation plots between the four subgroups and the high & low risk categories. **(C)** Survival curves for four subgroups. **(D-G)** PCA and t-SNE analyses of four subgroups. **(H-J)** Immune cell infiltration in four subgroups.

377 LIHC samples were regrouped according to the expression of NCLs, the cluster distribution function (CDF) had the smallest value when the clustering variable (k) was 4, and the consensus matrix contour was apparent (Fig 11A). Sankey plots showed that subgroups of C3 were more concentrated in low-risk regions, and subgroups of C2 were more concentrated in high-risk regions (Fig 11B). Correspondingly, the KM curve showed significant differences in the survival probability of different subgroups, with C3 having a better survival and C2 having the worst survival (Fig 11C). PCA and tSNE were used to identify whether the four clustered subgroups could better distinguish LIHC samples, which is beneficial for precision therapy for LIHC patients (Fig 11D–11G). In addition, analysis of the tumor microenvironment among subgroups showed that stromal, immune and estimation scores were higher in the C4 group (Fig 11H–11J).

**Fig 12:**
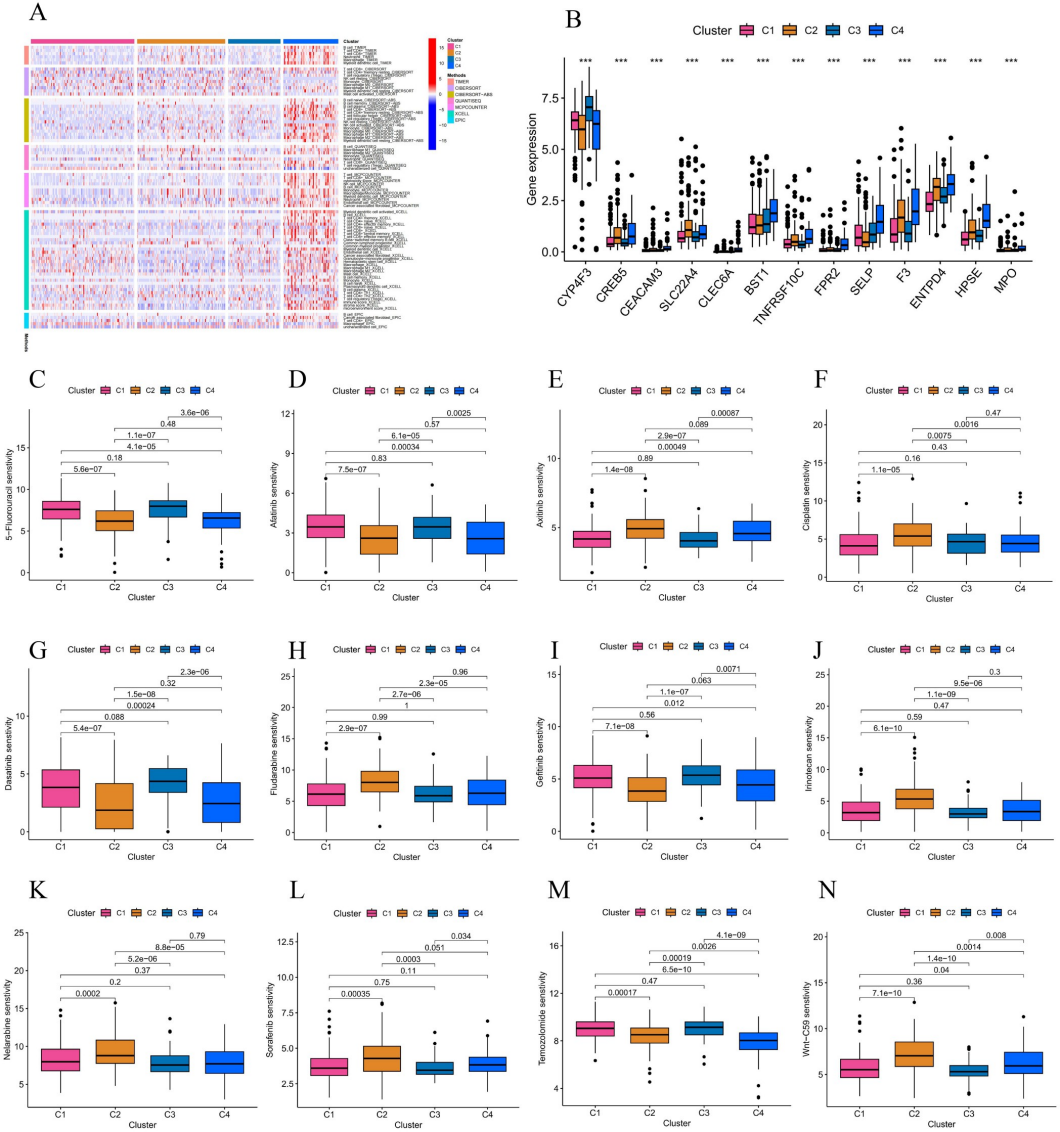
Immune cell relevance and drug sensitivity. **(A)** Immune cell correlations between subgroups. **(B)** Discrepancies in the expression of immune checkpoints between the four subgroups. **(C-N)** Variations in the sensitivity of various antineoplastic drugs among four subgroups.

According to the results of the heat map, the immune infiltration seemed to be more active in the C4 subgroup (Fig 12A), while the expression of different immune checkpoint molecules showed significant differences between subgroups (Fig 12B). Finally, drug susceptibility analyses showed differences in resistance to drugs in different subgroups of patients (Fig 12C–12N).

## 4. Discussion

As one of the malignant tumors with the highest morbidity and mortality rates worldwide, the formation, growth, and metastasis of LIHC are strongly associated with the organism’s inflammatory response [16]. Recent studies have shown that chronic inflammation in the liver can be triggered by hepatitis B virus (HBV) or hepatitis C virus (HCV) infection, and about 70% to 80% of LIHC patients have a history of hepatitis infection [17]. Surgical resection remains the mainstay of treatment for LIHC, but most patients have advanced disease at the time of initial diagnosis, with a resectable rate of only 20% to 30% [18]. Likewise, due to internal and external metastases of the tumor, many LIHC patients lose the chance of radical treatment [19]. Unfortunately, even after hepatectomy, the recurrence rate is as high as 70% after five years [20].

The emergence of immunotherapy has provided new treatment options for the treatment of patients with advanced LIHC. Immune checkpoint inhibitors have shown excellent therapeutic potential [21]. Sorafenib, gefitinib, etc., as effective molecularly targeted drugs against terminal liver tumors have also gained some clinical acceptance, and have been gradually trialed in LIHC patients around the world [22, 23]. In conclusion, the terrible therapeutic prospect of LIHC makes it necessary to establish reliable prognostic biomarkers and search for potential new drug targets.

Inflammation and immunity play a joint role in the constitution of the TME. The tumor’s inflammatory environment is regarded as the soil in which the cancer seed grows and develops, and some chronic inflammation triggers cancerous lesions through the accumulation of stimuli over a long period of time [24–26]. It is becoming increasingly clear that a complex population of immune cells, consisting of the innate immune system (including NK cells and macrophages) and the adaptive immune system, work together to identify and eliminate abnormal tumor cells [27, 28].

Neutrophils provide an essential function in the body’s innate immune system, and as a hallmark of cancer-associated inflammation, are often recruited in large numbers at cancer lesions [29, 30]. Notably, neutrophils have previously been identified as a possible treatment target for LIHC, involving anti-cancer immunity and direct enhancement of tumor cell survival, invasion and metastasis through stimulation of angiogenesis and degradation of extracellular matrix [31]. Taking this a step further, it is not difficult to speculate that NETs in the microenvironment of LIHC may accelerate tumor progression and promote the intricate drug-resistance mechanisms in LIHC.

The participation of LncRNAs in immune infiltration and prognostic treatment of LIHC has been highlighted in the previous studies. As an example, Wang et al. revealed that elevated LINC01225 expression led to poor prognosis in LIHC, while knockdown of LINC01225 decreased tumor cell proliferation and invasion [32]. In contrast, lncRNA miR503HG has the ability to inhibit metastasis of LIHC cells [33]. Other evidence suggests that lncRNAs contribute to abnormal LIHC metabolism and sustained proliferation by regulating the activity and expression of binding targets [34]. However, despite the progress made thus far, studies on NCLs are seldom mentioned.

To summarize, this study represents the first to develop an NCLs signature in LIHC, which not only helps to establish more effective biomarkers for the diagnosis and prognostic grading, but also provides a scientific and possible option for promoting individualized treatment of LIHC patients. This study five hub-NCLs (AC021188.1, AL158195.1, BACE1-AS, AL365361.1, MKLN1-AS) by regression filtering with transcriptome information in TCGA-LIHC. Further, we developed a risk model based on hub-NCLs along with categorized the patient sample into two groups: high- and low-risk, by the risk score. We also discovered that survivability was typically lower in the high-risk group, both in terms of OS and PFS. In particular, patients with elevated MKLN1-AS expression displayed a markedly worse prognosis, which is consistent with Guo et al.’s demonstration that MKLN1-AS is involved in liver tumor cell progression [35]. Intriguingly, independent prognostic analyses indicate that this risk model, similar to other clinical phenotypes, is one of the independent factor of LIHC. Additionally, we utilized the nomogram to forecast the samples’ prognosis at 1, 3, and 5 years. To distinguish biological functions and pathways between the high- and low-risk groups, we conducted GSEA analysis. As we suspected, multiple immune-related pathways were enriched, including the T-cell receptor, Wnt, PI3K-AKT, and ECM receptor-associated pathways. Remarkably, this is consistent with the mechanism that NE and MMP9 released during NETosis contribute to the destruction of the ECM, damaging the basement membrane and promoting cancer invasion and metastasis [36].

Stromal cell types and enrichment levels often determine TME properties, contributing to the response mechanisms of targeted immunotherapy [37]. In this study, we found substantial discrepancies in stromal and immune score between the high- and low- risk groups distinguished by NCLs, which may indicate that NETosis plays an essential role in tumor immunity. We also identified two risk typologies that behave dissimilarly in terms of immune cell abundance and function. In tumor immunity, M0 macrophages are in an inactive and immature state, which are believed to trigger the inflammatory response. The high-risk group showed significant overexpression of M0 macrophages, suggesting a potential link between their activity and the development and prognosis of LIHC [38]. In order to more precisely recognize tumor subtypes receiving targeted therapy and immunotherapy, all LIHC samples were classified into four subgroups based on consensus clustering. Drug sensitivity analyses were then performed to provide accurate treatment for patients with different types of LIHC.

The limitations of this current study must be acknowledged. Firstly, due to bias between data sets and lack of suitable survival data, we only selected TCGA data from a single source. Second, Although 18 reliable NRGs were collected from previous literature, as LIHC research progressed, it was necessary to optimize the risk model by adding new genes as the research progressed. Finally, there is a lack of in vivo and ex vivo experiments to test the underlying molecular mechanisms of hub NCLs.

## 5. Conclusion

In summary, we have constructed a novel signature based on the NCLs, which could help to predict the prognostic status of LIHC, as well as hopefully provide new insights to better elucidate the microenvironmental landscape and immunotherapy in LIHC.

## Supporting information

S1 Table A total of 18 NETosis-related genes were involved in the identification. (XLSX)
